# A novel interpretable machine learning framework integrating clinicopathological and radiomic features for early recurrence prediction in mass-forming intrahepatic cholangiocarcinoma

**DOI:** 10.1186/s40644-025-00987-6

**Published:** 2026-01-09

**Authors:** Xiao-li Deng, Chongze Yang, Lan-hui Qin, Xue-feng Lin, Fen Zhong, Jin-yuan Liao

**Affiliations:** 1 Department of Radiology, The First People’s Hospital of Yulin, Yulin, Guangxi Zhuang Autonomous Region 537000 People’s Republic of China; 2 Department of Radiology, The First Affiliated Hospital of Guangxi Medical University, Nanning, Guangxi Zhuang Autonomous Region 530021 People’s Republic of China; 3 Department of Radiology, Guangxi Hospital Division of the First Affiliated Hospital, Sun-Yat-Sen University, Nanning, Guangxi Zhuang Autonomous Region 530022 People’s Republic of China; 4 Key Laboratory of Early Prevention and Treatment for Regional High Frequency Tumor (Guangxi Medical University), Ministry of Education, Nanning, Guangxi Zhuang Autonomous Region 530021 People’s Republic of China

**Keywords:** Intrahepatic cholangiocarcinoma, Computed tomography, Radiomics, Machine learning, Prognosis

## Abstract

**Rationale and objectives:**

Early recurrence after curative resection remains a major determinant of poor prognosis in intrahepatic cholangiocarcinoma (ICC). Existing multimodal prediction models often lack interpretability due to feature interference during fusion. This study aimed to develop and externally validate an interpretable multimodal machine-learning model using a novel Independent Feature Selection and Consistent Integration (IFSCI) framework, combined with SHAP-based explanation to enhance transparency.

**Materials and methods:**

A total of 264 patients with mass-forming ICC who underwent radical resection were retrospectively enrolled from two centers. Clinical and CT-based radiomics features were independently selected within each modality using statistical testing and LASSO under the IFSCI design, ensuring modality-specific interpretability before consistent integration. Support vector machine (SVM), random forest (RF), and multilayer perceptron (MLP) algorithms were used to construct clinical, radiomics, and combined models. Model performance was evaluated using AUC, calibration curves, Brier score, and decision curve analysis (DCA). SHAP values were applied to provide global and case-level interpretability.

**Results:**

Six clinical and twenty radiomics features were retained. The MLP-based combined model demonstrated the best performance, with AUCs of 0.933 (training), 0.891 (internal validation), and 0.856 (external validation). Calibration and Brier scores confirmed good agreement, and DCA indicated clinical benefit across 10–30% threshold probabilities. SHAP visualizations revealed feature importance hierarchies and clarified the decision logic for individual predictions.

**Conclusions:**

By integrating IFSCI with SHAP-based explanations, this study provides a transparent, high-performance multimodal framework for early recurrence prediction in ICC, facilitating clinically trustworthy and interpretable decision support.

**Supplementary Information:**

The online version contains supplementary material available at 10.1186/s40644-025-00987-6.

## Introduction

Intrahepatic cholangiocarcinoma (ICC) is a primary malignant tumor originating from the epithelial cells of bile ducts located above the second-order intrahepatic bile ducts. It represents the second most common hepatic malignancy, accounting for approximately 10% to 15% of primary liver cancers [1, 2]. Compared to hepatocellular carcinoma, ICC is relatively uncommon; however, epidemiological studies suggest that the global incidence and mortality rates of ICC are steadily increasing, despite variations across different regions and countries [3]. The clinical presentation of ICC is nonspecific, with most patients remaining asymptomatic during the early stages. As the disease progresses, patients may experience symptoms such as weight loss, abdominal discomfort, jaundice, hepatomegaly, and palpable abdominal masses. Current evidence indicates that radical surgical resection offers the best chance for long-term survival in ICC patients [4, 5]. Unfortunately, ICC is highly aggressive and associated with poor prognosis. A systematic review reported that the 5-year overall survival rate following radical resection rarely exceeds 30%–35%, with a median overall survival of approximately 28 months [6]. Therefore, timely identification of patients at high risk for early recurrence is critical to improving patient prognosis.

Recently, considerable attention has been given to identifying risk factors for early recurrence of ICC; however, there is currently no consensus on the definition of early recurrence. Previous studies commonly defined early recurrence as recurrence within two years post-surgery, a criterion similar to that used for hepatocellular carcinoma (HCC) [7, 8]. However, ICC generally carries a worse prognosis compared to HCC, with most recurrences occurring within the first two years after surgical resection. Therefore, defining early recurrence with a two-year threshold may not be appropriate for ICC patients [2]. A growing body of research indicates that recurrence within one year is strongly associated with poorer outcomes, suggesting that defining early recurrence as recurrence within one year might be more suitable for ICC patients [9–11]. Hence, accurately predicting recurrence within the first year after surgery holds significant importance for improving patient prognosis and guiding clinical decision-making.

With the continuous advancement of computer technologies, radiomics and machine learning have emerged as novel diagnostic tools in oncology, making prognostic prediction for ICC increasingly reliable [12]. Radiomics, as a research hotspot in imaging science, utilizes computer-assisted techniques to quantitatively extract subtle imaging features that are imperceptible to the human eye [13]. This approach allows for a shift from subjective interpretation to objective analysis, showing remarkable potential in predicting ICC prognosis based on imaging data [14]. Moreover, machine learning enables researchers to identify prognostically relevant features from complex clinical and pathological datasets, thereby facilitating outcome prediction for ICC patients [15]. Given that a single data modality may not fully capture the heterogeneity of ICC, integrating multimodal data may offer a more comprehensive solution. Machine learning models developed through the integration of radiomics, clinical, and pathological features can significantly enhance early recurrence prediction, yielding synergistic clinical benefits that surpass the value of any single modality alone [16].

However, most current studies suffer from limitations in their multimodal model construction strategies. Typically, features from various modalities—such as clinical, pathological, and radiomics—are combined first, followed by a unified feature selection process and model development [17]. This approach presents two major drawbacks: (1) The selected features in the final combined model may differ greatly—or even entirely—from those used in independently constructed unimodal models, thereby reducing both the comparability between unimodal and multimodal models and the interpretability of the combined model. (2) Imbalances in feature quantity across modalities—for instance, a large number of radiomics features versus relatively few clinical or pathological features—can result in the exclusion of clinically meaningful modality-specific features due to inter-feature interference during joint selection [18, 19].

Overall, such a strategy weakens both the interpretability of multimodal models and their comparability with unimodal counterparts. An alternative approach—independently selecting features within each modality and then consistently integrating them into the final combined model—may enhance both model interpretability and cross-model comparability.

Furthermore, it is important to note that machine learning faces an inherent limitation in clinical applications: it is often referred to as a “black-box algorithm” due to its lack of interpretability [20, 21]. This opaque computational process reduces the level of trust and acceptance among clinicians, thereby restricting the widespread adoption of machine learning in clinical practice. However, for complex clinical tasks such as tumor prognosis, accurate and transparent model interpretation is both crucial and highly challenging. Previous studies have attempted to translate machine learning results into clinical practice using tools such as nomograms, which help visualize model predictions and facilitate clinical application to some extent. Nevertheless, these tools do not fundamentally assist clinicians in understanding the decision-making process of machine learning models [15]. In recent years, the emergence of Shapley additive explanations (SHAP), an interpretation method grounded in game theory, has offered a novel solution to this challenge [22]. By calculating SHAP values, this method assigns to each feature its marginal impact on the prediction, clarifying its importance within the model and elucidating the logic behind its decision.

To address feature-interference in existing multimodal models, we propose the Independent Feature Selection and Consistent Integration (IFSCI) strategy. IFSCI independently screens and retains each modality’s key features, then consistently integrates them into a unified model to predict early recurrence risk in ICC patients, thereby enhancing model comparability and interpretability. Furthermore, we apply SHAP‐based explainability analysis at both global and local levels to dissect the model’s decision‐making process, identify the most influential features, and ultimately improve the model’s transparency, interpretability, and clinical credibility.

## Materials and methods

**Fig. 1:**
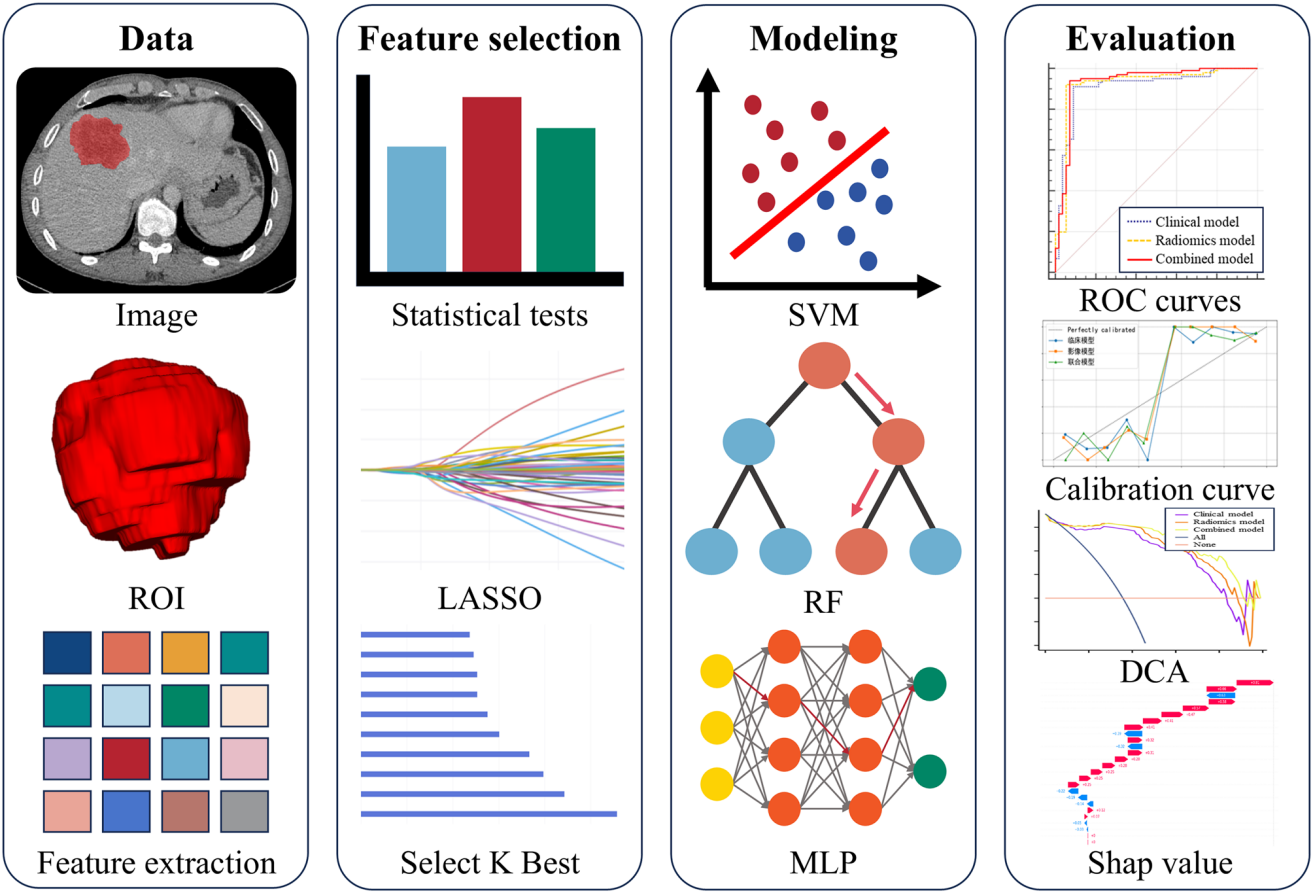
Overall study flowchart. ROI: Region of Interest. LASSO: Least Absolute Shrinkage and Selection Operator. SVM: Support Vector Machine. RF: Random Forest. MLP: Multilayer Perceptron. ROC: Receiver Operating Characteristic. DCA: Decision Curve Analysis

We followed the TRIPOD (Transparent Reporting of a Multivariable Prediction Model for Individual Prognosis or Diagnosis) guidelines to report the development and validation of the multivariable prediction model [23]. This retrospective study was approved by the ethics committee of hospital, with informed consent waived. The overall study flowchart is presented in Fig. 1.

### Patients and clinical characteristics

This multicenter cohort comprised ICC cases diagnosed between January 2014 and December 2023 at two Hospital. All patients underwent radical surgical resection, and histopathological analysis confirmed the diagnosis of ICC.

The inclusion criteria were as follows: (1) intrahepatic mass-forming cholangiocarcinoma with a solitary lesion larger than 1 cm in diameter; (2) radical surgical resection performed; (3) postoperative pathological confirmation of ICC with complete pathological data; and (4) All patients underwent both non‑contrast and contrast‑enhanced liver CT scans within one month prior to surgery. The exclusion criteria were: (1) incomplete clinical data; (2) presence of distant metastasis; (3) absence of qualified preoperative CT images; (4) receipt of arterial embolization or other interventional therapies before CT examination; and (5) lack of follow-up records.

Clinical data collected included age, sex, history of alcohol consumption, and presence of liver cirrhosis. Preoperative tumor markers such as serum AFP, CA19-9, CA125, and CEA were also recorded. Pathological data were retrospectively collected, including tumor differentiation grade, lymph node metastasis status, microvascular invasion, surgical margin status, and Ki-67 expression levels from immunohistochemical analysis.

### Follow-up surveillance

All patients included in this study underwent radical surgical resection and were regularly followed up at the two participating medical centers. The study endpoint was the occurrence of early recurrence after radical resection for ICC, which was defined as recurrence within 12 months postoperatively. All enrolled patients followed a standardized postoperative monitoring protocol, with evaluations scheduled at 1, 3, 6, and 12 months post-surgery. Follow-up assessments included serological tumor markers such as CA19-9, AFP, and CEA, as well as abdominal ultrasound, contrast-enhanced CT, and contrast-enhanced MRI. The data were reviewed in December 2024.

### CT image acquisition

CT images were acquired using multidetector spiral CT scanners. At Institution 1, the scanners included the Siemens SOMATOM Force, Siemens SOMATOM Definition Flash, GE LightSpeed VCT, and GE Revolution 256-slice spiral CT, while Institution 2 utilized the Siemens Sensation 64 CT and GE Discovery CT750. All patients underwent both non-contrast and contrast-enhanced liver CT scans within one month before surgery. The protocol involved an initial conventional plain scan of the liver, followed by contrast-enhanced scans.

### Image segmentation and radiomics feature extraction

The CT images from the arterial and portal venous phases were imported into ITK-SNAP (version 3.8). Radiologist 1 (with 12 years of experience) manually delineated the tumor volumes of interest (VOIs) on the arterial and portal venous phase images, respectively. All VOIs were confirmed by a second radiologist (with 15 years of experience), and any disputes were resolved through discussion involving a third radiologist (with 24 years of experience). In addition, after more than 3 months, radiologist 1 randomly selected 20 patients again to delineated the ROI. When the extracted features intra-class correlation coefficients (ICC) were greater than 0.75, the radiomics feature extraction showed good consistency.

All CT images were standardized prior to radiomics extraction. The arterial- and portal-phase images were resampled to an isotropic voxel spacing of 1 × 1 × 1 mm³, the window width and level were harmonized across scanners. These preprocessing steps were applied uniformly to minimize scanner-dependent variability.

Quantitative radiomics features were extracted using the Python package pyradiomics. A total of 1,051 features were computed from the delineated VOIs based on CT images from both the arterial and portal venous phases. The selected variables comprised first‑order statistics and shape descriptors, along with texture metrics extracted from gray‑level co‑occurrence (GLCM), run‑length (GLRLM), size‑zone (GLSZM), dependence (GLDM), and neighboring gray‑tone difference (NGTDM) matrices, as well as higher‑order features.

### Feature selection and model construction

Patient data from Center One (*n* = 196) were stratified using an 8:2 ratio, resulting in a training set of 157 cases and an internal validation set of 39 cases. Data from Center Two (*n* = 68) were used as an external test set. The radiologist performed two intragroup segmentations, and the resulting ICC values were greater than 0.75, indicating that the radiomics feature extraction had satisfactory consistency.

All feature-selection procedures and model training were performed exclusively within the training set to avoid information leakage. The internal validation set and the external test cohort were not involved in any step of feature selection or model training; instead, they served solely as independent datasets for model evaluation. This design ensured a fully leakage-free workflow and allowed unbiased assessment of model generalizability.

### Clinical feature

For clinical features, categorical variables were compared between groups using the chi-square test, while continuous variables were assessed using either the Mann–Whitney U test or the independent-samples t-test. Only variables showing statistically significant differences between the two groups were subsequently incorporated into the model-building process.

### Radiomics feature

Radiomics feature selection was performed using a structured, stage-wise reduction workflow to address the high dimensionality and multicollinearity inherent in the extracted features. First, all radiomics features were standardized using Z-score normalization. A Mann–Whitney U test was then employed as an initial univariate filtering step to remove features without detectable discriminatory ability between groups, thereby improving computational efficiency for subsequent multivariate procedures. Second, the Least Absolute Shrinkage and Selection Operator (LASSO) regression was applied to further refine the feature set by resolving multicollinearity. Through penalized coefficient shrinkage based on an optimized tuning parameter (λ), LASSO reduced the feature space to a subset with stable and non-redundant predictive contributions. In this workflow, LASSO served as an intermediate selection step to derive an interpretable, low-correlation feature group suitable for downstream model development. Third, SelectKBest was used to standardize the dimensionality of the final feature set. The number of retained features was set to K = 10 for each imaging phase, following the commonly recommended 10:1 sample-to-feature ratio in radiomics research. This design ensured an appropriate balance between feature complexity and available sample size, thereby reducing overfitting risk and enhancing model robustness.

### Model construction

In this study, clinical pathological features and radiomics features were separately input into Support Vector Machine (SVM), Random Forest (RF), and Multilayer Perceptron (MLP) algorithms to construct clinical and radiomics models for predicting early recurrence of ICC. Subsequently, to enhance comparability and interpretability between unimodal and multimodal approaches, clinical and radiomics features were merged and fed into a single framework to assess the risk of early ICC recurrence.

### Evaluation of model performance

The performance of the predictive models was evaluated using the area under the receiver operating characteristic curve (AUC), along with sensitivity, specificity, accuracy, Youden index, F1-score, and Brier score. Calibration curves and decision curve analysis (DCA) were employed to assess the stability and clinical benefit of the models, respectively. Threshold probabilities between 10% and 30% were selected because this range reflects clinically meaningful decision points in postoperative ICC management, where recurrence probabilities below 10% rarely alter follow-up strategies, whereas probabilities above 20–30% often trigger intensified surveillance or consideration of adjuvant treatment. The DeLong test was used to evaluate performance distinctions between models.

### SHAP value: model interpretation based on game theory

To better understand and interpret the fundamental decision rules of the machine learning model, this study incorporated SHAP values to elucidate the decision-making process. SHAP, a post hoc explanation method grounded in game theory, computes the marginal contribution of each feature to the model output. Consequently, it provides clear and intuitive linear explanations for the impact of individual features, thereby enabling both global and local interpretability of the model [22].

### Statistical analysis

Radiomics feature extraction was performed using the open-source pyradiomics package in Python (version 3.7). All data analyses were conducted using R software (version 4.3.3) and Python (version 3.7). A P value of < 0.05 was considered statistically significant.

## Results

### Patient

Of the 264 ICC cases enrolled, 196 came from Institution 1 (126 with recurrence, 70 without) and 68 from Institution 2 (46 with recurrence, 22 without) (Table 1, and Supplementary Materials 1).

**Table 1:** Clinical characteristics of ICC patients in the training set

Clinical characteristics	Recurrence (*n* = 126)	No recurrence (*n* = 70)	Statistics	*P*
Age (years)	55.79 ± 10.73	55.30 ± 10.53	-0.842	0.401
Ki67			37.759	**0**
≤ 20	27 (21.43%)	46 (65.71%)		
>20	99 (78.57%)	24 (34.29%)		
Drinking history			1.084	0.298
Yes	49 (61.11%)	22 (31.43%)		
No	77 (38.89%)	48 (68.57%)		
Diabetes			0.03	0.863
Yes	21 (16.67%)	11 (15.71%)		
No	105 (83.33%)	59 (84.29%)		
AST			3.567	0.059
≤ 40	69 (54.76%)	48 (68.57%)		
>40	57 (45.24%)	22 (31.43%)		
ALT			0.246	0.62
≤ 40	71 (56.35%)	42 (60.00%)		
>40	55 (43.65%)	28 (40.00%)		
GGT			4.927	**0.026**
≤ 60	46 (36.51%)	37 (52.86%)		
>60	80 (63.49%)	33 (47.14%)		
TBIL(umol/L)			0.528	0.467
0	101 (80.16%)	53 (75.71%)		
1	25 (19.84%)	17 (24.29%)		
CEA(ng/mL)			12.492	**0**
< 5	71 (56.35%)	57 (81.43%)		
≥ 5	55 (43.65%)	13 (18.57%)		
CA125(U/mL)			3.083	0.079
< 35	82 (65.08%)	54 (77.14%)		
≥ 35	44 (34.92%)	16 (22.86%)		
CA199(U/mL)			0.261	0.609
< 34	60 (47.62%)	36 (51.43%)		
≥ 34	66 (52.38%)	34 (48.57%)		
CA153(U/mL)			6.827	**0.009**
< 28	89 (70.63%)	61 (87.14%)		
≥ 28	37 (29.37%)	9 (12.86%)		
AFP(ng/mL)			8.764	**0.003**
< 20	93 (73.81%)	64 (91.43%)		
≥ 20	33 (26.19%)	6 (8.57%)		
Clonorchis sinensis infection			2.226	0.136
Yes	69 (54.76%)	24 (34.29%)		
No	57 (45.24%)	46 (65.71%)		
Peritumoral bile duct stones			1.043	0.307
Yes	9 (7.14%)	8 (11.43%)		
No	117 (92.86%)	62 (88.57%)		
Peritumoral bile duct dilatation			0.037	0.848
Yes	63 (50.00%)	34 (48.57%)		
No	63 (50.00%)	36 (51.43%)		
Liver capsular retraction			0.002	0.966
Yes	68 (53.97%)	38 (54.29%)		
No	58 (46.03%)	32 (45.71%)		
Vascular tumor thrombus			0.064	0.8
Yes	40 (31.75%)	21 (30.00%)		
No	86 (68.25%)	49 (70.00%)		
Enlarged lymph nodes			1.227	0.268
Yes	58 (46.03%)	38 (54.29%)		
No	68 (53.97%)	32 (45.71%)		
Tumor location			2.637	0.268
Perihilar type	25 (19.84%)	21 (30.00%)		
Peripheral type	82 (65.08%)	39 (55.71%)		
both perihilar and peripheral regions	19 (15.08%)	10 (14.29%)		
Tumor morphology			0.53	0.467
Regular	67 (53.17%)	41 (58.57%)		
Irregular	59 (46.83%)	29 (41.43%)		
Maximum tumor diameter (cm)	6.71 ± 3.11	6.00 ± 2.80	-0.062	0.951
CT plain scan			4.132	**0.042**
Homogeneous density	22 (17.46%)	21 (30.00%)		
Heterogeneous density	104 (82.54%)	49 (70.00%)		
Arterial phase characteristics			0.504	0.777
Isodensity or hypodensity	44 (34.92%)	21 (30.00%)		
Heterogeneous hyperdensity	14 (11.11%)	8 (11.43%)		
Rim-like hyperdensity	68 (53.97%)	41 (58.57%)		
Enhancement pattern			6.08	0.108
Type 1	38 (30.16%)	33 (47.14%)		
Type 2	48 (38.10%)	21 (30.00%)		
Type 3	31 (24.60%)	11 (15.71%)		
Type 4	9 (7.14%)	5 (7.15%)		
Histological differentiation			0.757	0.384
Low	53 (42.06%)	25 (35.71%)		
Moderate - high	73 (57.94%)	45 (64.29%)		
CK19			0.008	0.931
Negative	2 (1.59%)	1 (1.43%)		
Positive	124 (98.41%)	69 (98.57%)		
CK7			0.857	0.355
Negative	14 (11.11%)	11 (15.71%)		
Positive	112 (88.89%)	59 (84.29%)		
P53			1.334	0.248
Negative	35 (27.78%)	25 (35.71%)		
Positive	91 (72.22%)	45 (64.29%)		
Liver cirrhosis			0.157	0.692
Yes	13 (10.32%)	6 (8.57%)		
No	113 (89.68%)	64 (91.43%)		
MVI			2.11	0.348
M0	75 (59.52%)	41 (58.57%)		
M1	45 (35.71%)	22 (31.43%)		
M2	6 (4.77%)	7 (10.00%)		

Type 1: Typical rim enhancement with gradual inward filling; Type 2: Progressive hypoenhancement; Type 3: Arterial phase rim enhancement with portal phase washout; Type 4: Heterogeneous persistent hyperenhancement

### Feature selection

For clinical pathological features, among the 196 patients from Institution 1, six clinical features—namely, Ki67, GGT, CEA, CA153, AFP, and CT plain scan—showed statistically significant differences with respect to the early recurrence of ICC. Thus, the clinical and combined models were each built upon these six features (Table 1).

**Fig. 2:**
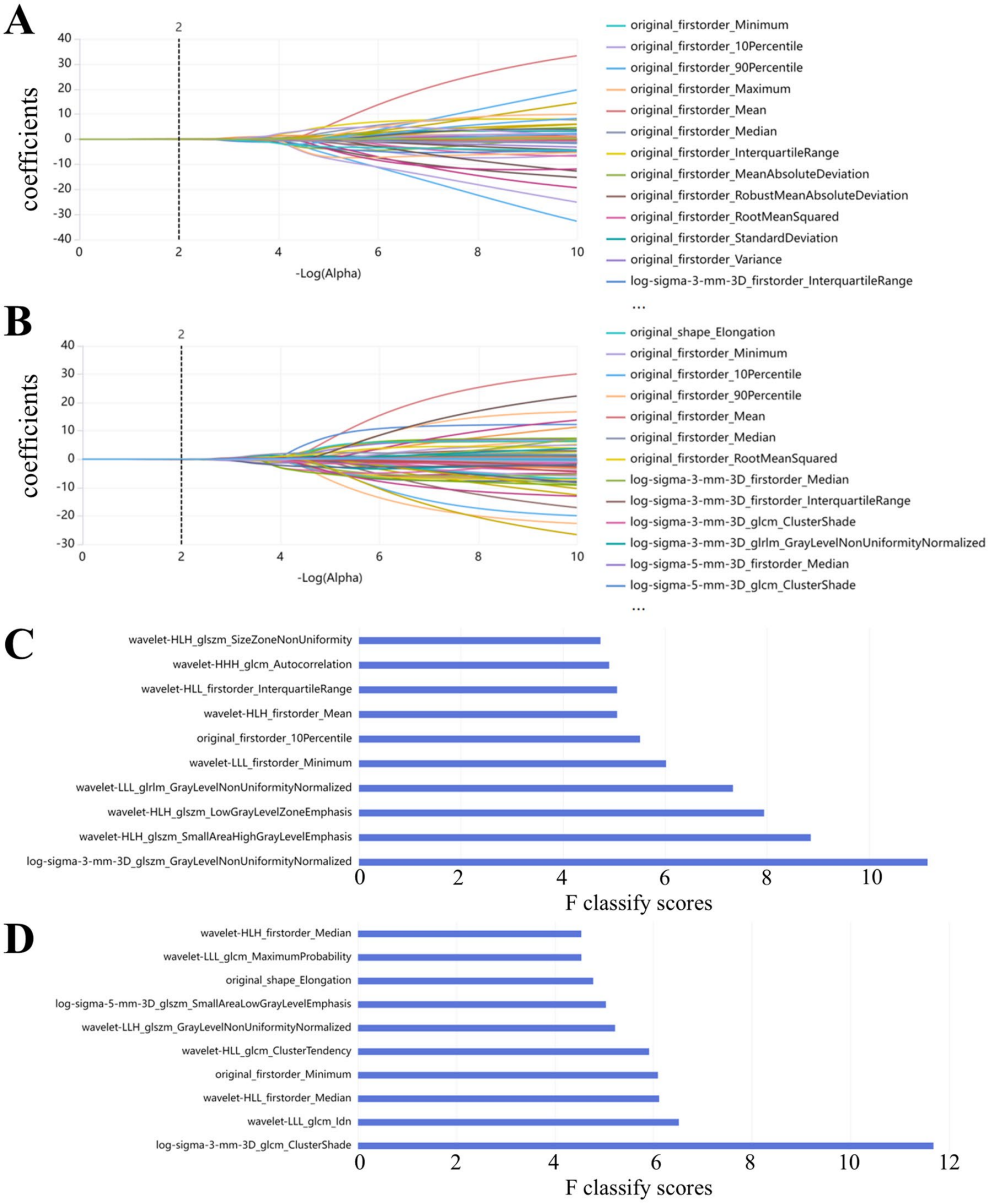
Feature selection processes using LASSO and K-best methods. Panels (**A**–**B**) illustrate the LASSO process for feature selection in the arterial phase (**A**) and portal venous phase (**B**), respectively. Panels (**C**–**D**) display the K-best feature selection process for the arterial phase (**C**) and portal venous phase (**D**), respectively

Regarding radiomics features, an initial screening using the Mann–Whitney U test identified 88 arterial phase CT radiomics features and 123 portal venous phase CT radiomics features (*p* < 0.05). Subsequently, the LASSO was employed to further refine these features, ultimately selecting 23 arterial phase and 26 portal venous phase CT radiomics features. The LASSO coefficient path illustrating this process is shown in Fig. 2.

Furthermore, given that the total number of cases in the training and test sets was 196, and to avoid overfitting, the SelectKBest was applied to further reduce the number of features, standardizing the total number of radiomics features to 20. Ultimately, the top 10 features from each of the arterial and portal venous phase feature sets were selected for constructing the radiomics and combined models (Fig. 2).

### Performance comparison of different models

In this study, based on the 20 selected radiomics features and 6 clinical features, radiomics, clinical, and combined radiomics–clinical models were constructed using SVM, RF, and MLP algorithms (Table 2 and Supplementary Materials 2).

**Table 2:** Comprehensive feature list included in the model

Types of features	Features list
Radiomic features in arterial phase	wavelet-HLH glszm SizeZoneNonUniformity
	wavelet-HHH glcm Autocorrelation
	wavelet-HLL firstorder InterquartileRange
	wavelet-HLH firstorder Mean
	original firstorder 10Percentile
	wavelet-LLL firstorder Minimum
	wavelet-LLL glrlm GrayLevelNonUniformityNormalized
	wavelet-HLH glszm LowGrayLevelZoneEmphasis
	wavelet-HLH glszm SmallAreaHighGrayLevelEmphasis
	log-sigma-3-mm-3D glszm GrayLevelNonUniformityNormalized
Radiomic features of portal venous phase	wavelet-HLH firstorder Median
	wavelet-LLL glcm MaximumProbability
	original shape Elongation
	log-sigma-5-mm-3D glszm SmallAreaLowGrayLevelEmphasis
	wavelet-LLH glszm GrayLevelNonUniformityNormalized
	wavelet-HLL glcm ClusterTendency
	original firstorder Minimum
	wavelet-HLL firstorder Median
	wavelet-LLL glcm ldn
	log-sigma-3-mm-3D glcm ClusterShade
Clinical characteristics	Ki67
	GGT
	CEA
	CA153
	AFP
	CT plain scan

Regarding the clinical model, integrating six clinicopathological indicators, the predictive algorithm demonstrated strong accuracy in forecasting early ICC recurrence. Notably, the MLP algorithm achieved superior performance in the training set, with an AUC of 0.911 (95% CI: 0.855–0.950), compared to the SVM algorithm (AUC = 0.826, 95% CI: 0.758–0.882) and the RF algorithm (AUC = 0.815, 95% CI: 0.745–0.872). This performance trend was consistently observed in both the internal and external validation sets (Table 3).

**Table 3:** Model diagnostic performance

Algorithm	Dataset	Model	AUC (95% CI)	Sensitivity	Specificity	Comparison with combined model
SVM	Training set	Clinical model	0.826 (0.758–0.882)	84.16	83.93	Z = 1.693, *P* = 0.0904
		Radiomics model	0.834 (0.767–0.889)	87.13	82.14	Z = 1.312, *P* = 0.1895
		Combined model	0.896 (0.837–0.939)	88.12	85.71	
	Internal validation set	Clinical model	0.771 (0.609–0.890)	76.00	78.57	Z = 0.676, *P* = 0.4988
		Radiomics model	0.811 (0.654–0.919)	84.00	78.57	Z = 0.332, *P* = 0.7400
		Combined model	0.851 (0.701–0.945)	88.00	85.71	
	External validation set	Clinical model	0.712 (0.590–0.816)	76.09	72.73	Z = 1.112, *P* = 0.2662
		Radiomics model	0.796 (0.681–0.884)	80.43	72.73	Z = 0.194, *P* = 0.8464
		Combined model	0.813 (0.700–0.897)	84.78	81.82	
RF	Training set	Clinical model	0.815 (0.745–0.872)	86.14	73.21	Z = 1.254, *P* = 0.2100
		Radiomics model	0.853 (0.788–0.905)	90.10	80.36	Z = 0.501, *P* = 0.6163
		Combined model	0.867 (0.803–0.916)	92.08	80.36	
	Internal validation set	Clinical model	0.774 (0.612–0.892)	84.00	78.57	Z = 0.413, *P* = 0.6796
		Radiomics model	0.791 (0.631–0.905)	88.00	85.71	Z = 1.342, *P* = 0.1795
		Combined model	0.826 (0.671–0.928)	88.00	78.57	
	External validation set	Clinical model	0.716 (0.594–0.819)	78.26	72.73	Z = 0.734, *P* = 0.4632
		Radiomics model	0.772 (0.654–0.865)	80.43	72.73	Z = 0.299, *P* = 0.7653
		Combined model	0.801 (0.687–0.888)	84.78	81.82	
MLP	Training set	Clinical model	0.911 (0.855–0.950)	91.09	91.07	Z = 0.620, *P* = 0.5354
		Radiomics model	0.925 (0.872–0.961)	92.08	94.64	Z = 0.209, *P* = 0.8341
		Combined model	0.933 (0.881–0.966)	94.06	92.86	
	Internal validation set	Clinical model	0.811 (0.654–0.919)	84.00	78.57	Z = 0.728, *P* = 0.4668
		Radiomics model	0.823 (0.667–0.926)	84.00	85.71	Z = 0.614, *P* = 0.5395
		Combined model	0.891 (0.750–0.968)	88.00	85.71	
	External validation set	Clinical model	0.753 (0.633–0.850)	78.26	72.73	Z = 1.503, *P* = 0.1328
		Radiomics model	0.811 (0.698–0.896)	82.61	81.82	Z = 0.617, *P* = 0.5374
		Combined model	0.856 (0.749–0.929)	84.78	81.82	

The radiomics model was constructed by combining 10 arterial phase radiomics features with 10 portal venous phase radiomics features. The radiomics model surpassed the clinical model in performance across all three machine learning algorithms (SVM, RF, and MLP). Notably, the MLP algorithm achieved the best predictive performance, with AUCs of 0.925 (95% CI: 0.872–0.961) in the training set, 0.823 (95% CI: 0.667–0.926) in the internal validation set, and 0.811 (95% CI: 0.698–0.896) in the external validation set (Table 3).

In this study, a combined model was further developed by integrating 20 imaging features and 6 clinical features using three different machine learning algorithms (SVM, RF, and MLP) to predict early recurrence of ICC. Among the combined models constructed with these algorithms, diagnostic performance across training, internal validation, and external validation cohorts surpassed that of the unimodal clinical and radiomics models. Notably, the combined model developed using the MLP algorithm achieved the best predictive performance, with AUCs of 0.933 (95% CI, 0.881–0.966) in the training set, 0.891 (95% CI, 0.750–0.968) in the internal validation set, and 0.856 (95% CI, 0.749–0.929) in the external validation set (Table 3).

### Evaluation and application of calibration curves, DCA

**Fig. 3:**
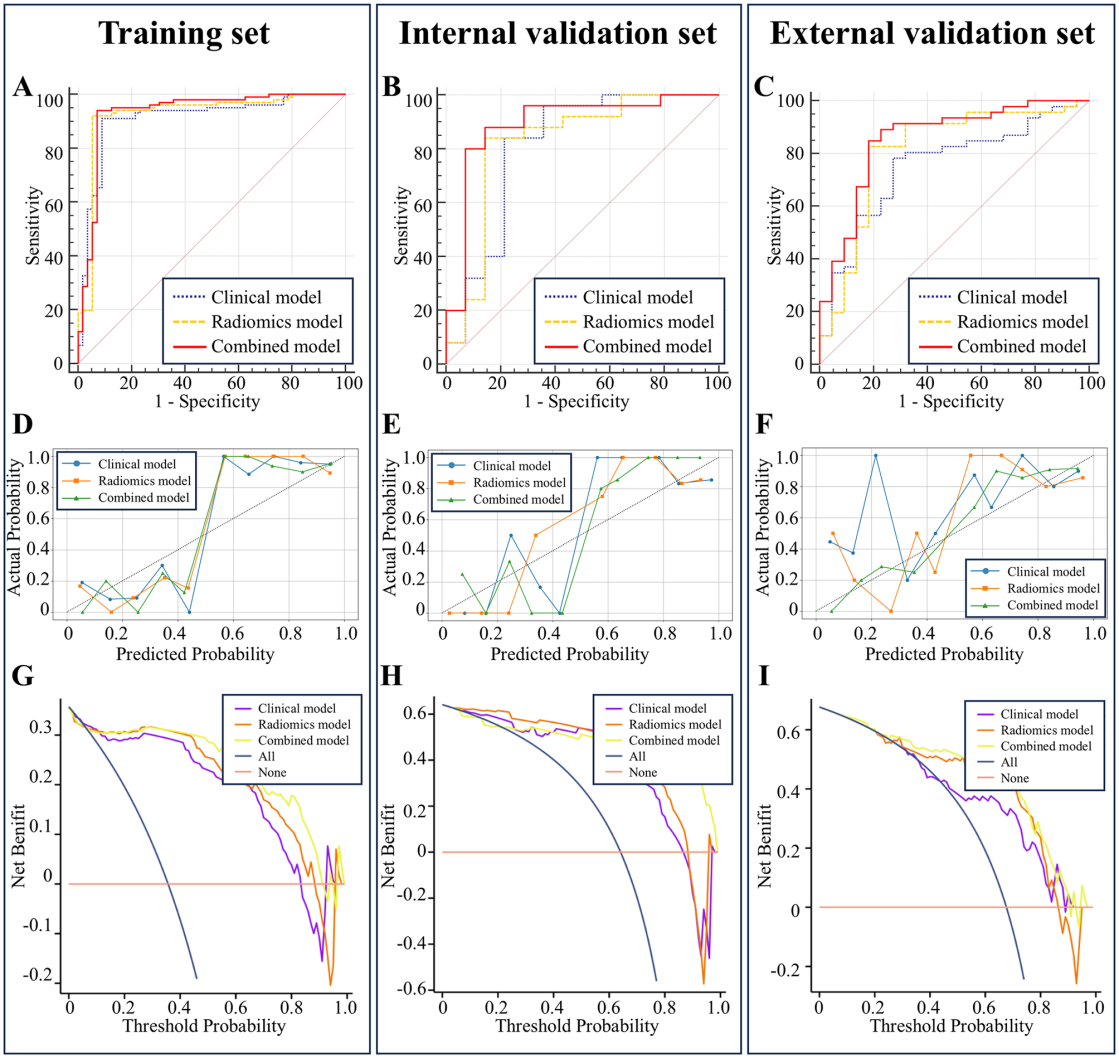
Composite figure of MLP diagnostic performance. Panels (**A**–**C**) display the ROC curves (AUC), Panels (**D**–**F**) show the calibration curves, and Panels (**G**–**I**) illustrate the decision curve analysis (DCA)

Calibration curves further demonstrated a high level of concordance between the predicted values and the observed outcomes, underscoring the reliability of the model’s predictions. Moreover, DCA revealed that, compared to solely empirical clinical judgment, the combined model conferred a significant net benefit for patients across a broader range of risk thresholds (Fig. 3).

### SHAP values: global and local interpretability

**Fig. 4:**
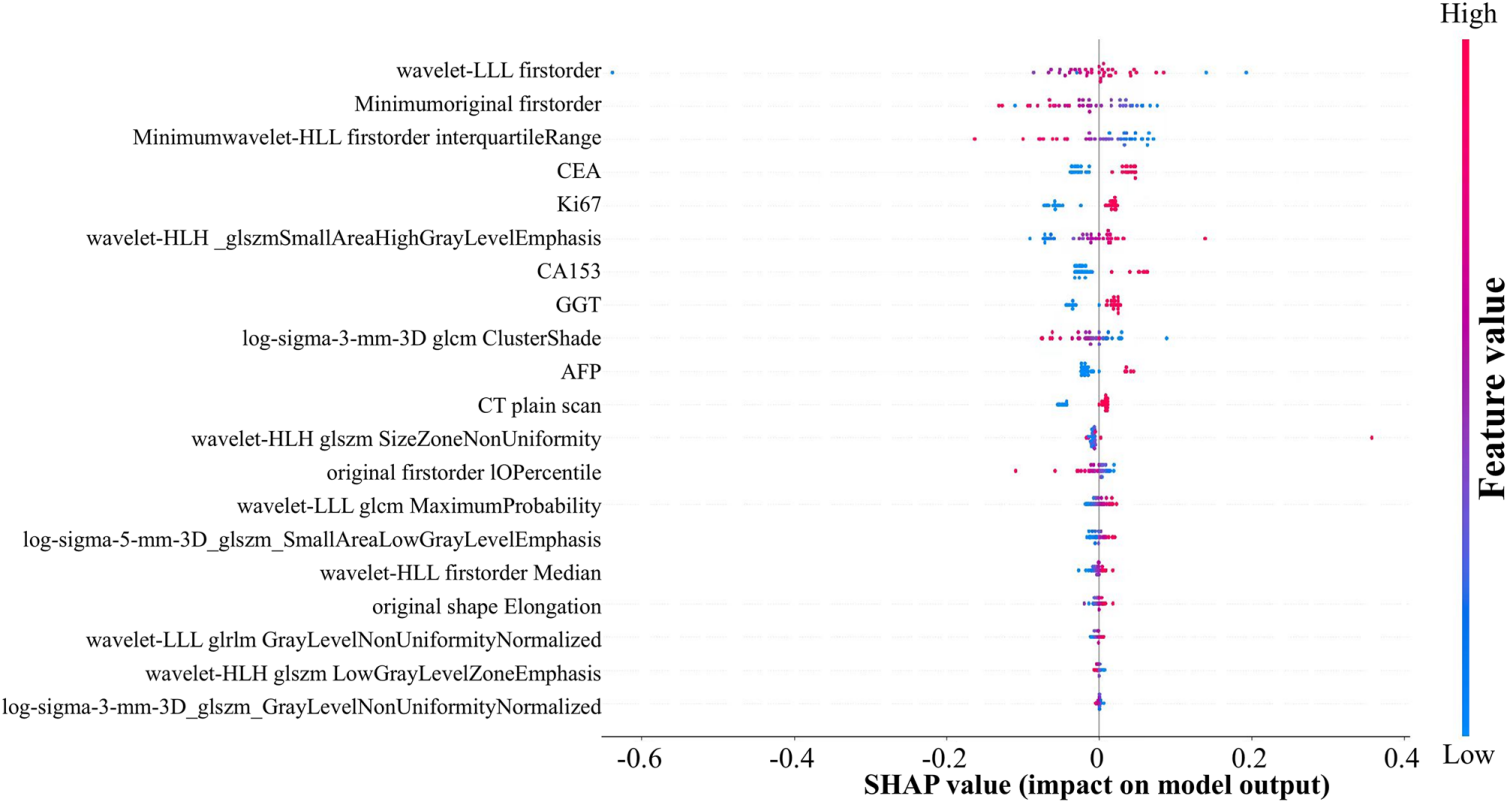
SHAP bee swarm plot – global interpretation of the MLP-based combined model

To enhance the interpretability of the model, we employed SHAP values to provide both global and local explanations for the MLP-based combined model that demonstrated the highest predictive performance. SHAP analysis was performed exclusively on the final 26 features. The SHAP bee-swarm plot offers a global interpretation by quantifying each feature’s contribution to the model output. In the plot, the vertical axis lists feature names in descending order of their importance to the model prediction. Each dot represents the specific value of the feature for an individual sample; the color intensity and horizontal position of the dot indicate the magnitude of the feature’s value and whether its impact on the prediction is positive or negative. Furthermore, the density of dots reflects the frequency of feature values within the dataset (Fig. 4).

**Fig. 5:**
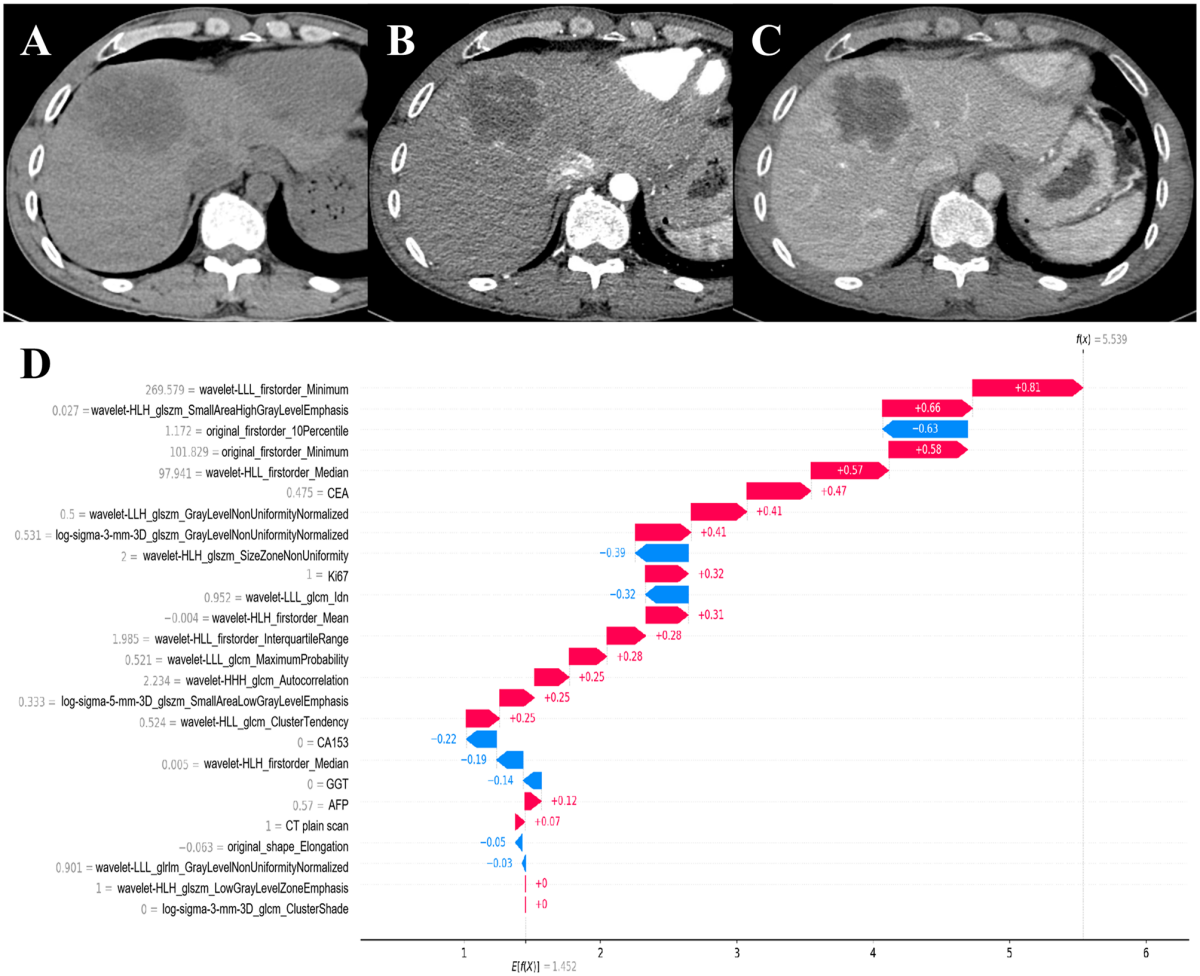
Local explanation provided by SHAP values for a specific case. Panels (**A**–**C**) display the plain scan, arterial phase, and portal venous phase images of an ICC patient with early recurrence. Panel (**D**) shows the SHAP waterfall plot for this patient, which indicates a positive cumulative SHAP value, suggesting that the model predicted early recurrence

In addition, SHAP values facilitate detailed local interpretation for individual cases. In this context, the length of each feature bar denotes the magnitude of its contribution to the prediction, while the direction of the bar indicates whether the feature increases (positive contribution) or decreases (negative contribution) the likelihood of early recurrence. The sum of all SHAP values for the features constitutes the overall SHAP total for that case; a positive total suggests that the model is inclined to predict a high risk of early recurrence, whereas a negative total indicates a low risk (Fig. 5).

Integrating SHAP values with the machine learning model facilitates clinicians’ understanding of the decision-making process from both global and local perspectives. This integration enhances the model’s transparency and clinical interpretability, thereby increasing the credibility and practical utility of its predictive outcomes. Ultimately, it contributes to more precise identification of ICC patients at high risk of early recurrence and provides robust evidence to support precision medicine in clinical practice.

## Discussion

In this study, a machine learning model was established using an IFSCI approach. Separate clinical models, radiomics models, and a multimodal combined model were developed based on clinical-pathological features and radiomics features. The results demonstrated that both the clinical and radiomics models exhibited robust predictive performance for early recurrence in ICC, While fusing clinical and radiomic feature sets further improved predictive efficacy. Among the three machine learning algorithms evaluated, the MLP model showed superior predictive performance, a finding that was corroborated by the calibration curves and DCA. Overall, this method provided high inter-model comparability and interpretability, and the inclusion of SHAP values further facilitated both global and local interpretation of the machine learning models.

Recognizing the prognostic relevance of clinical features in ICC, we selected six clinical parameters for analysis—Ki67, GGT, CEA, CA153, AFP, and findings on plain CT scans—demonstrated significant correlations with early recurrence after ICC surgery, which aligns with the bulk of existing literature. Specifically, Ki-67 is a nuclear protein closely related to cellular proliferation, and its expression level is positively correlated with tumor aggressiveness. Several studies have identified Ki-67 as an independent predictor of poor prognosis in ICC [24, 25]. Moreover, Ki-67 is increasingly regarded as a potential therapeutic target in malignancies; for example, the CDK inhibitor Dinaciclib has been shown to suppress ICC growth by targeting and downregulating Ki-67 expression [26]. In addition, silencing the long non-coding RNA (lncRNA) CASC15 effectively reduces Ki-67 levels, thereby hindering ICC progression [27]. Gamma-glutamyl transpeptidase (GGT), a liver function marker, is closely linked to the occurrence and progression of hepatic tumors. Elevated GGT levels have been associated with vascular invasion, lymph node involvement, incomplete tumor encapsulation, and advanced ICC based on higher Child–Pugh grading. Furthermore, studies have demonstrated that patients with high GGT levels exhibit a median recurrence-free survival of only 6 months, compared to 12 months in patients with low GGT [28]. Carcinoembryonic antigen (CEA) is a widely used marker for gastrointestinal malignancies; its increased levels are significantly associated with higher tumor burden, metastasis, and worse prognosis in ICC [29]. Additionally, high expression levels of AFP and CEA in tumor tissues are significantly correlated with decreased survival in ICC patients [25]. CA153, a mucin-like glycoprotein broadly expressed in various cancers and linked to tumor cell proliferation and invasion, suggests a higher degree of malignancy; however, its prognostic value in ICC requires further investigation [30]. As a conventional marker for hepatocellular carcinoma, AFP has been reported to correlate with tumor invasiveness, differentiation, and poor prognosis in ICC patients. Elevated AFP is also strongly associated with microvascular invasion (MVI) in ICC [31], and serum AFP levels have been shown to independently predict overall survival (OS) in patients with HBV-related ICC [32]. Lastly, a heterogeneous density observed on plain CT scans is considered a high-risk factor for early ICC recurrence. This heterogeneous density reflects the complex tumor heterogeneity arising from high tumor aggressiveness, which leads to changes such as tumor necrosis and hemorrhage [33].

In this study, radiomic predictors of early ICC recurrence in this study were drawn primarily from first‑order statistics, shape, and texture descriptors. Specifically, first-order statistical features (e.g., Mean, Median, Interquartile Range) indicate variations in the distribution of gray-level intensities within the tumor, reflecting intratumoral density heterogeneity; texture features (e.g., those derived from GLCM, GLRLM, and GLSZM) elucidate the complexity of the tumor’s microstructure and suggest spatial heterogeneity; and shape features (e.g., Elongation) reveal irregularities or invasiveness in tumor growth [34, 35]. Overall, these features collectively capture the imaging characteristics of ICC, which include high-density heterogeneity, intricate internal structure, and irregular growth patterns, thereby showing a close association with the risk of early recurrence.

Early identification of adverse outcomes and the improvement of overall survival in cancer patients have long been key objectives for medical researchers. Thanks to comprehensive analyses of multimodal data, the early detection of high-risk ICC recurrence is now achievable [18]. Currently, methods for integrating multimodal data are generally categorized into early, intermediate, and late fusion strategies. Among these, early (data-level) fusion and feature-level fusion are widely favored because they impose lower requirements on the data and the models [36, 37]. Numerous studies have applied radiomics for predicting early postoperative recurrence in ICC, consistently demonstrating that multimodal combined models offer superior predictive performance. However, most studies tend to focus primarily on predictive accuracy while neglecting model interpretability. In many cases, a strategy of combining features before feature selection (i.e., early fusion) is used; this approach not only risks a dimensionality explosion but also results in significant discrepancies between the features utilized in unimodal models and those incorporated into the multimodal combined model, thereby making it challenging to isolate the individual impact of each unimodal feature. Consequently, this diminishes both the interpretability of the multimodal model and its comparability with unimodal models. The IFSCI strategy employed in this study effectively addresses these issues by first independently selecting features from each modality and then consistently integrating them. This not only enhances model interpretability but also improves comparability between multimodal and unimodal models. Moreover, this strategy provides a clearer foundation for model interpretation and clinical decision-making, thereby increasing clinicians’ trust in complex predictive models.

In terms of machine learning algorithms, we concurrently evaluated the performance of Multilayer Perceptron (MLP), Random Forest (RF), and Support Vector Machine (SVM). The MLP consistently demonstrated superior predictive performance across all models, which may be attributed to its strong nonlinear mapping capabilities and superior generalization performance [38]. Although RF and SVM are widely used in medical image analysis, the robust learning ability of MLP—derived from its artificial neural network architecture—renders it more effective in capturing the latent nonlinear relationships between clinical and radiomics features in complex datasets. In real-world scenarios, data variables often exhibit both linear and nonlinear associations. As a feedforward neural network, MLP consists of an input layer, one or more hidden layers, and an output layer, with each layer comprising multiple neurons; it employs nonlinear activation functions to perform complex feature transformations [39]. However, MLP’s high data requirements necessitate future validation on larger external cohorts to strengthen its generalizability and robustness.

The interpretability of machine learning models is paramount in the medical field, as it directly influences clinicians’ acceptance of predictive outcomes and the models’ clinical translatability. To enhance model interpretability, this study employed SHAP values for model explanation [22]. SHAP’s advantage lies in its game theory-based, post hoc interpretation approach, which can uncover complex patterns learned by predictive models, going beyond the limitations of simpler modeling methods. The integration of SHAP values not only enables visualization of the model prediction process but also provides patient-specific, localized explanations, thereby making it easier for clinicians to understand the rationale behind the model’s outputs. Overall, SHAP values effectively elucidate the workings of machine learning models and enhance their applicability in clinical practice. However, it should be noted that SHAP explains the patterns learned by the model rather than directly interpreting the underlying sample features; as a result, explaining outliers that deviate significantly from the overall trend remains challenging—a difficulty inherent to machine learning predictions. Future studies involving larger sample sizes for model training and the incorporation of deep learning-based interpretation methods may help overcome these limitations.

In summary, this study significantly improved the models’ predictive performance and interpretability by implementing an IFSCI strategy in conjunction with SHAP value integration. This approach elucidated the contribution of individual features within the multimodal framework for predicting early postoperative recurrence in ICC. Among the algorithms evaluated, the MLP model demonstrated superior predictive power, likely owing to its robust nonlinear mapping capability. However, One notable limitation is the study’s relatively small sample size, no comparison was made between different model interpretation methods, and variability in CT scanner acquisition parameters may introduce fluctuations in radiomic feature extraction. Future studies should recruit larger, multicenter cohorts and establish standardized imaging acquisition and preprocessing protocols to minimize heterogeneity, thereby facilitating clinical translation of the model and enabling precise postoperative management and individualized treatment for ICC patients. In addition, clinical variables were selected using univariate statistical testing rather than embedded or wrapper machine-learning methods. This choice was made to maintain clinical interpretability, although it does not account for multivariate interactions. A further limitation concerns the radiomics feature selection strategy. The Mann–Whitney U-test may exclude features that become informative only through multivariate interactions, and the fixed K value in the SelectKBest step may constrain the flexibility of dimensionality reduction. Future studies should incorporate sensitivity analyses using alternative selection approaches and varying K values to assess the robustness of the selected feature set. Future studies with larger cohorts will further explore ML-based feature-selection strategies.

## Conclusions

The IFSCI approach combined with SHAP values facilitates the development of highly interpretable multimodal machine learning models for predicting early recurrence following ICC surgery. Compared to traditional unimodal prediction models, the multimodal combined model further enhances predictive performance, with the MLP algorithm demonstrating superior outcomes due to its robust nonlinear processing capabilities. Future research should focus on validation through multicenter studies and large sample cohorts to further promote clinical translation, ultimately advancing precise postoperative management and individualized treatment for ICC patients.

## Supplementary Information

Below is the link to the electronic supplementary material.

Supplementary Material 1

Supplementary Material 2

Supplementary Material 3

Supplementary Material 4

## Data Availability

Not applicable.

## Abbreviations

ICC: Intrahepatic cholangiocarcinoma
CT: Computed Tomography
SVM: Support vector machine
RF: Random forest
MLP: Multilayer perceptron
ROI: Region of interest
LR: Logistic regression
ROC: Receiver Operating Characteristic
AUC: Area under the curve
DCA: Decision curve analysis
SHAP: Shapley additive explanations
LASSO: Least Absolute Shrinkage and Selection Operator
