# Supplementary material for: A novel interpretable machine learning framework integrating clinicopathological and radiomic features for early recurrence prediction in mass-forming intrahepatic cholangiocarcinoma
Source: Cancer Imaging. 2026 Jan 9;26:23. doi: 10.1186/s40644-025-00987-6 (PMC12882180; doi:10.1186/s40644-025-00987-6)
Supplement: Supplementary file 2 — Supplementary Material 2 [file 40644_2025_987_MOESM2_ESM.docx]

**Supplementary Material 1: Clinical Data of Patients in the External Validation Dataset.**

| Clinical characteristics | Total (n = 68) | No recurrence (n = 22) | Recurrence (n = 46) |
| --- | --- | --- | --- |
|  |  |  |  |
| Age (years) | 56.60 ± 11.34 | 53.77 ± 8.46 | 57.96 ± 12.34 |
| Ki67 |  |  |  |
| ≤20 | 27 (39.71) | 17 (77.27) | 10 (21.74) |
| ＞20 | 41 (60.29) | 5 (22.73) | 36 (78.26) |
| Drinking history |  |  |  |
| 0 | 45 (66.18) | 14 (63.64) | 31 (67.39) |
| 1 | 23 (33.82) | 8 (36.36) | 15 (32.61) |
| Diabetes |  |  |  |
| 0 | 62 (91.18) | 19 (86.36) | 43 (93.48) |
| 1 | 6 (8.82) | 3 (13.64) | 3 (6.52) |
| AST |  |  |  |
| ≤40 | 39 (57.35) | 12 (54.55) | 27 (58.70) |
| ＞40 | 29 (42.65) | 10 (45.45) | 19 (41.30) |
| ALT |  |  |  |
| ≤40 | 43 (63.24) | 11 (50.00) | 32 (69.57) |
| ＞40 | 25 (36.76) | 11 (50.00) | 14 (30.43) |
| GGT |  |  |  |
| ≤60 | 42 (61.76) | 10 (45.45) | 32 (69.57) |
| ＞60 | 26 (38.24) | 12 (54.55) | 14 (30.43) |
| TBIL, n(%) |  |  |  |
| 0 | 32 (47.06) | 7 (31.82) | 25 (54.35) |
| 1 | 36 (52.94) | 15 (68.18) | 21 (45.65) |
| CEA, n(%) |  |  |  |
| 0 | 39 (57.35) | 8 (36.36) | 31 (67.39) |
| 1 | 29 (42.65) | 14 (63.64) | 15 (32.61) |
| CA125, n(%) |  |  |  |
| <35 | 46 (67.65) | 15 (68.18) | 31 (67.39) |
| ≥35 | 22 (32.35) | 7 (31.82) | 15 (32.61) |
| CA-199, n(%) |  |  |  |
| <34 | 32 (47.06) | 10 (45.45) | 22 (47.83) |
| ≥34 | 36 (52.94) | 12 (54.55) | 24 (52.17) |
| CA153, n(%) |  |  |  |
| <28 | 50 (73.53) | 14 (63.64) | 36 (78.26) |
| ≥28 | 18 (26.47) | 8 (36.36) | 10 (21.74) |
| AFP, n(%) |  |  |  |
| <20 | 49 (72.06) | 12 (54.55) | 37 (80.43) |
| ≥20 | 19 (27.94) | 10 (45.45) | 9 (19.57) |
| Clonorchis sinensis infection |  |  |  |
| Yes | 64 (94.12) | 20 (90.91) | 44 (95.65) |
| No | 4 (5.88) | 2 (9.09) | 2 (4.35) |
| Peritumoral bile duct stones |  |  |  |
| Yes | 59 (86.76) | 20 (90.91) | 39 (84.78) |
| No | 9 (13.24) | 2 (9.09) | 7 (15.22) |
| Peritumoral bile duct dilatation |  |  |  |
| Yes | 32 (47.06) | 11 (50.00) | 21 (45.65) |
| No | 36 (52.94) | 11 (50.00) | 25 (54.35) |
| Liver capsular retraction |  |  |  |
| Yes | 39 (57.35) | 12 (54.55) | 27 (58.70) |
| No | 29 (42.65) | 10 (45.45) | 19 (41.30) |
| Vascular tumor thrombus |  |  |  |
| Yes | 39 (57.35) | 13 (59.09) | 26 (56.52) |
| No | 29 (42.65) | 9 (40.91) | 20 (43.48) |
| Enlarged lymph nodes |  |  |  |
| Yes | 42 (61.76) | 16 (72.73) | 26 (56.52) |
| No | 26 (38.24) | 6 (27.27) | 20 (43.48) |
| Tumor location |  |  |  |
| Perihilar type | 37 (54.41) | 13 (59.09) | 24 (52.17) |
| Peripheral type | 7 (10.29) | 2 (9.09) | 5 (10.87) |
| both perihilar and peripheral regions | 24 (35.29) | 7 (31.82) | 17 (36.96) |
| Tumor morphology |  |  |  |
| Regular | 19 (27.94) | 7 (31.82) | 12 (26.09) |
| Irregular | 49 (72.06) | 15 (68.18) | 34 (73.91) |
| Maximum tumor diameter (cm) | 5.96 ± 2.75 | 5.96 ± 2.75 | 5.96 ± 2.75 |
| CT plain scan |  |  |  |
| Homogeneous density | 8 (11.76) | 1 (4.55) | 7 (15.22) |
| Heterogeneous density | 48 (70.59) | 16 (72.73) | 32 (69.57) |
| CT plain scan | 12 (17.65) | 5 (22.73) | 7 (15.22) |
| Arterial phase characteristics |  |  |  |
| Isodensity or hypodensity | 29 (42.65) | 7 (31.82) | 22 (47.83) |
| Heterogeneous hyperdensity | 35 (51.47) | 13 (59.09) | 22 (47.83) |
| Rim-like hyperdensity | 4 (5.88) | 2 (9.09) | 2 (4.35) |
| Enhancement pattern |  |  |  |
| Type 1 | 16 (23.53) | 6 (27.27) | 10 (21.74) |
| Type 2 | 43 (63.24) | 13 (59.09) | 30 (65.22) |
| Type 3 | 7 (10.29) | 2 (9.09) | 5 (10.87) |
| Type 4 | 2 (2.94) | 1 (4.55) | 1 (2.17) |
| Histological differentiation |  |  |  |
| Low | 21 (30.88) | 3 (13.64) | 18 (39.13) |
| Moderate - high | 47 (69.12) | 19 (86.36) | 28 (60.87) |
| CK19 |  |  |  |
| Negative | 6 (8.82) | 4 (18.18) | 2 (4.35) |
| Positive | 62 (91.18) | 18 (81.82) | 44 (95.65) |
| CK7 |  |  |  |
| Negative | 13 (19.12) | 4 (18.18) | 9 (19.57) |
| Positive | 55 (80.88) | 18 (81.82) | 37 (80.43) |
| P53 |  |  |  |
| Negative | 25 (36.76) | 8 (36.36) | 17 (36.96) |
| Positive | 43 (63.24) | 14 (63.64) | 29 (63.04) |
| Liver cirrhosis |  |  |  |
| Yes | 56 (82.35) | 21 (95.45) | 35 (76.09) |
| No | 12 (17.65) | 1 (4.55) | 11 (23.91) |
| MVI |  |  |  |
| M0 | 45 (66.18) | 13 (59.09) | 32 (69.57) |
| M1 | 20 (29.41) | 7 (31.82) | 13 (28.26) |
| M2 | 3 (4.41) | 2 (9.09) | 1 (2.17) |
