# Supplementary material for: A novel interpretable machine learning framework integrating clinicopathological and radiomic features for early recurrence prediction in mass-forming intrahepatic cholangiocarcinoma
Source: Cancer Imaging. 2026 Jan 9;26:23. doi: 10.1186/s40644-025-00987-6 (PMC12882180; doi:10.1186/s40644-025-00987-6)
Supplement: Supplementary file 4 — Supplementary Material 4 [file 40644_2025_987_MOESM4_ESM.docx]

**Feature Selection**

Patients from Center One (n = 196) were randomly divided into a training set (n = 157) and an internal validation set (n = 39) using stratified sampling (ratio 4:1). Data from Center Two (n = 68) served as an independent external test set.

To strictly avoid information leakage, all feature-selection procedures were performed exclusively within the training set, whereas the internal validation set and external test set were used solely for model evaluation and did not participate in any feature selection or model training steps.

The aim of radiomics feature selection was to (i) reduce dimensionality, (ii) improve model stability and generalizability, (iii) minimize redundancy, and (iv) prevent overfitting while ensuring computational efficiency. The feature-selection pipeline consisted of Z-score normalization, Mann–Whitney U tests, LASSO regression, and SelectKBest, performed as follows:

(1) Z-score normalization

Radiomic features extracted from the training set were standardized using Z-score normalization to improve comparability across samples and stabilize subsequent statistical analyses.

(2) Mann–Whitney U test

Each radiomic feature underwent univariate screening using the Mann–Whitney U test to identify features that differed significantly between the early-recurrence and non-recurrence groups.

Features with p < 0.05 were retained for further selection.

This step yielded:

①88 arterial-phase features

②123 portal-venous–phase features

These preliminarily selected features likely reflect biologically relevant tumor heterogeneity associated with ICC recurrence.

(3) LASSO regression

Least Absolute Shrinkage and Selection Operator (LASSO) regression was then applied to the U-test–filtered features within the training set. LASSO introduces an L1-regularization penalty, which shrinks coefficients of weak predictors to zero and thereby performs embedded feature selection.

The regularization parameter (α) controls the sparsity of the coefficient set. A larger α produces stronger penalization and yields a more compact feature subset. In this study, the α parameter was set to 0.01, resulting in:

①23 arterial-phase features

②26 portal-venous–phase features

The LASSO coefficient path plot illustrates how coefficients of individual features evolve as α increases. As α grows, coefficients of less informative features shrink toward zero, enabling an interpretable and robust selection of meaningful predictors.

(4) SelectKBest dimensionality reduction

Because radiomics studies often include far more features than samples, excessive dimensionality may lead to overfitting. The commonly recommended sample-to-feature ratio of approximately 10:1 was therefore adopted.

Given 196 samples from Center One used in model development, we restricted the total radiomic feature count to 20 features.

Using SelectKBest, the top K features were ranked according to their F-statistics. Based on this criterion:

①10 arterial-phase and

②10 portal-venous–phase features

SelectKBest enables selection of the most informative and non-redundant features, optimizing the predictive capability while limiting model complexity.

Summary：The complete feature-selection process was: Training set only → Z-score normalization → Mann–Whitney U test → LASSO regression → SelectKBest → Final 20 radiomic features. Internal and external validation sets did not participate in any step of feature selection, ensuring a rigorous leakage-free workflow.

**Model Training and Hyperparameter Optimization**

Building on these configurations, we adopted a standardized and fully reproducible model‐training pipeline across all algorithms. For the SVM classifier, an RBF kernel was selected due to its ability to capture nonlinear boundaries commonly observed in radiomic data. A comprehensive grid search was performed over a predefined hyperparameter space (C: [0.1, 1, 10, 100, 1000]; gamma: [0.001, 0.01, 0.1, 1, “scale”, “auto”]) within a nested cross-validation framework, yielding the optimal combination of C = 10 and gamma = 0.01. This ensured a balance between model flexibility and generalization performance.

For the Random Forest model, we implemented an ensemble of 500 decision trees, with no explicit constraint on maximum depth to allow each tree to grow sufficiently deep to capture high-order feature interactions. The mtry parameter was set to the square root of the total number of input features, following standard heuristics that enhance decorrelation among trees and stabilize variance. Bootstrap sampling was applied at each iteration to further improve robustness.

For the MLP classifier, we constructed a two-hidden-layer neural architecture with 128 and 64 neurons, respectively. ReLU activation was applied to all hidden units to promote sparse representations and mitigate vanishing gradients. A Sigmoid activation was used in the output layer to generate calibrated probabilities for binary classification. The network was optimized using Adam (learning rate = 0.001), trained for up to 200 epochs with a batch size of 32. Early stopping based on validation loss was incorporated to prevent overfitting. The random seed was fixed at 42 to ensure deterministic behavior across runs. Moreover, weight initialization and data-shuffling schemes were kept consistent to minimize stochastic variability.
